# Patient Reported Outcomes in Large Vessel Vasculitides

**DOI:** 10.1007/s11926-020-00979-4

**Published:** 2021-01-28

**Authors:** Joanna Robson, Sarah Mackie, Catherine Hill

**Affiliations:** 1 Centre for Health and Clinical Research, University of the West of England, Bristol, UK; 2 Rheumatology Department, University Hospitals Bristol and Weston NHF Foundation Trust, Bristol, UK; 3 Vascular Rheumatology, Leeds Institute of Rheumatic and Musculoskeletal Medicine, University of Leeds, Leeds, UK; 4 Rheumatology Department, Leeds Teaching Hospital NHS Trust, Chapel Allerton Hospital, Leeds, UK; 5 Rheumatology Unit, The Queen Elizabeth Hospital, Woodville, South Australia Australia; 6 Division of Medicine, University of Adelaide, Adelaide, South Australia Australia

**Keywords:** Giant cell arteritis, Takayasu arteritis, Patient reported outcome, Clinical trials, Outcomes, Large vessel vasculitis

## Abstract

**Purpose of Review:**

The goal of this paper is to review current and future uses of patient-reported outcomes in large vessel vasculitis. The large vessel vasculitides comprise Giant Cell Arteritis and Takayasu arteritis; both are types of systemic vasculitis which affect the larger blood vessels. Patient-reported outcomes (PROs) capture the impact of these diseases on health-related quality of life.

**Recent Findings:**

Generic PROs such as the SF-36 are currently used to compare HRQOL of people with GCA and TAK within clinical trials and observational studies and to make comparisons with the general population and HRQoL in other diseases. The development of a disease-specific PRO for GCA is currently underway. Beyond clinical trials, there is much interest in the use of PROs within routine clinical care, particularly E-PROs for remote use.

**Summary:**

Further work will be needed to complete the development of disease-specific PROs for people with large vessel vasculitis and to establish feasibility, acceptability, and utility of E-PROs.

## Introduction

The 2012 International Chapel Hill Consensus Conference for nomenclature of the systemic vasculitides defines the large vessel vasculitides (LVVs) as giant cell arteritis (GCA) and Takayasu arteritis (TA) [1]. These are the primary systemic vasculitides and are characterized by inflammation of the aorta, and its proximal branches accompanied by systemic inflammation with elevation in several circulating cytokines including interleukin (IL)-6. Other systemic diseases, including IgG4 disease, may also cause inflammation of the large vessels including the aorta, but these are considered secondary vasculitides and are outside the scope of this review as their clinical manifestations in other organ systems must also be considered. The challenge with large vessel vasculitis (LVV) is that inflammation of the aorta and its proximal branches may be asymptomatic or present with seemingly vague symptoms such as malaise, fatigue, weight loss, fever, or myalgia. Consequently diagnosis is frequently delayed [2] and monitoring may be challenging. The potential for patient-reported outcomes to enhance care for these patients is hitherto unexplored.

GCA is the commonest form of systemic vasculitis in people over the age of 50; it is estimated that by 2050, more than 3 million people will have been diagnosed with GCA in Europe, North America, and Oceania [3]. GCA classically presents with headache, jaw claudication, flu-like symptoms, and inflammatory pain and stiffness in the shoulders and hips (polymyalgia rheumatica, present in 50%) [4]. There is a risk of blindness in 20% of cases if untreated [4, 5]. Glucocorticoids have been the mainstay of treatment for GCA for decades. Adjuvant immunosuppression using the IL6-inhibitor tocilizumab (TCZ) has been shown in clinical trials to reduce the risk of GCA relapse and increases the chance of sustained glucocorticoid-free remission at 1 year [6]. On this basis TCZ is now licensed and recommended as an adjuvant treatment for GCA [7, 8]. Importantly the pivotal TCZ trial, GiACTA, included patient-reported outcomes as secondary outcome measures. In contrast trials of methotrexate for GCA gave equivocal results. A conditional recommendation for methotrexate as adjuvant therapy was made [7] on the basis of an individual patient data analysis of the three trials. If a common set of patient-reported outcomes had been measured in each of these trials, this might have given a clearer answer on the clinical utility of MTX in GCA.

**Fig. 1:**
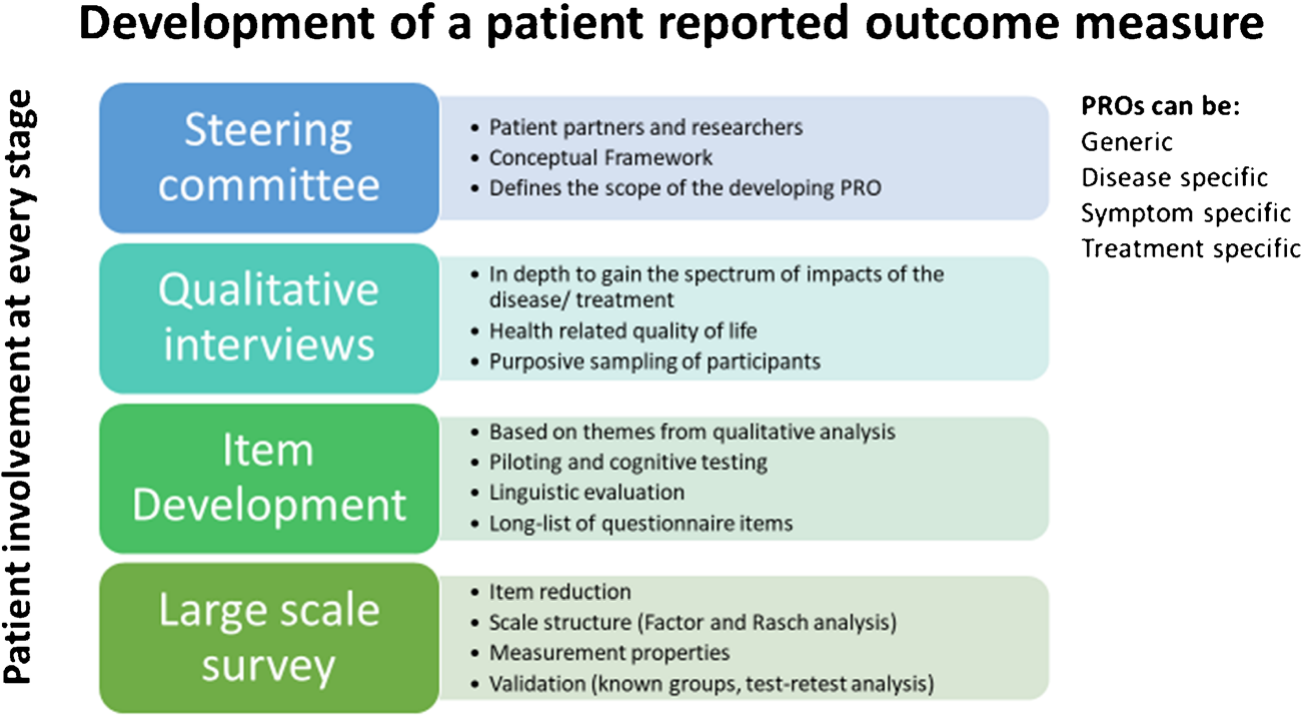
Development of a patient reported outcome measure

Takayasu arteritis (TAK) is a rarer form of LVV which predominantly affects women of childbearing age and involves the aorta and its main branches and the pulmonary arteries [9]. Takayasu arteritis therefore differ from giant cell arteritis in terms of age of onset, distribution of arterial involvement, and symptoms. Weight loss, fever, and fatigue are key constitutional symptoms, while vascular inflammation and occlusion lead to pain, claudication, and tissue loss [10]. Patients with Takayasu are also treated with glucocorticoids but in combination with non-biological disease-modifying agents from the start (as opposed to in GCA where glucocorticoids alone are the standard first line treatments); in relapsing or refractory patients, TCZ or anti-tumor necrosis factor inhibitors are also considered [8].

### What Is the Impact on Health-Related Quality of Life?

Having GCA can impact on health-related quality of life due to symptoms (e.g., pain, visual disturbance and musculoskeletal symptoms), glucocorticoids adverse effects, and loss of feeling “normal”) [11]. Patients are concerned about delays in getting a diagnosis and also fear going blind [11]. There is an association between vision-related quality of life scores and global health-related quality of life in people with GCA [12]. People with GCA ranked the following topics as most important to them: “losing sight in both eyes permanently,” “having intense or severe pain,” and “feeling weak, tired or exhausted” [13]. There is also the psychological impact of weighing up fear of treatment with glucocorticoids versus fear of going blind [14]. Dealing with a chronic illness that runs an unpredictable course frequently demands “work” on the part of the patient who has to plan their life around an uncertain short- and long-term future; coming to terms with requirements for higher dosages of glucocorticoids during active disease is part of that work in GCA [15]. Interestingly, patients with GCA who received TCZ in the GIACTA trial reported clinically meaningful improvements in overall health-related quality of life and fatigue compared with those receiving glucocorticoids alone [16]. After 1 year, HRQoL in the treatment group was the same as age and gender matched controls and exceeded normal values in some domains [16]. This finding challenges preconceptions that fatigue and depression, which are characterized by elevation in IL-6 [17], are an inevitable part of systemic inflammatory diseases such as GCA and TAK; monitoring health-related quality of life may be an efficient way of assessing how well the underlying inflammatory disease is being controlled by treatment, particularly since measurement of acute phase markers in TCZ-treated patients tends to be clinically uninformative.

People living with TAK have higher levels of anxiety and depression and greater physical limitations than healthy people [18]. People in Turkey who have been diagnosed with TAK have poorer health-related quality of life than healthy controls; with scores comparable to people with other inflammatory rheumatic conditions such as rheumatoid arthritis [19]. People living in the USA with TAK were found to have poorer HRQoL if they were taking more immunosuppressants (this is likely to be related to increased severity of disease) and if they had active disease or were older age [20].

#### Recent Findings

##### How Is Health-Related Quality of Life Measured in the Large Vessel Vasculitides?

Measurement of HRQoL scores in LVV has mainly been based on generic patient reported outcomes (PROs), specifically the Short-Form 36 (SF-36) [21]. The benefits of the SF-36 are that it is a well-recognized and validated outcome measure [21] and allows comparison between people withLVV, other conditions and general population controls, for example, in the HRQoL report from the GIACTA study [16]. Symptom-specific patient-reported outcomes, for example, the FACIT-Fatigue outcome, have also been used to compare one aspect of HRQoL [22] within a randomized controlled trial [16]. Studies in people with TAK have also used the SF36 to compare with the general population and other diseases [19].

Because generic PROs have been designed to be relevant across diseases and populations, there is the potential that face and content validity of the PRO to people with the disease under study is reduced [23]. This lack of specificity could reduce the ability to detect differences in state between people with the disease and in the same person over time [23]. In GCA, for example, using known groups analyses, the SF-36 does not detect differences between patients with and without visual loss or systemic involvement for example [24].

**Fig. 2:**
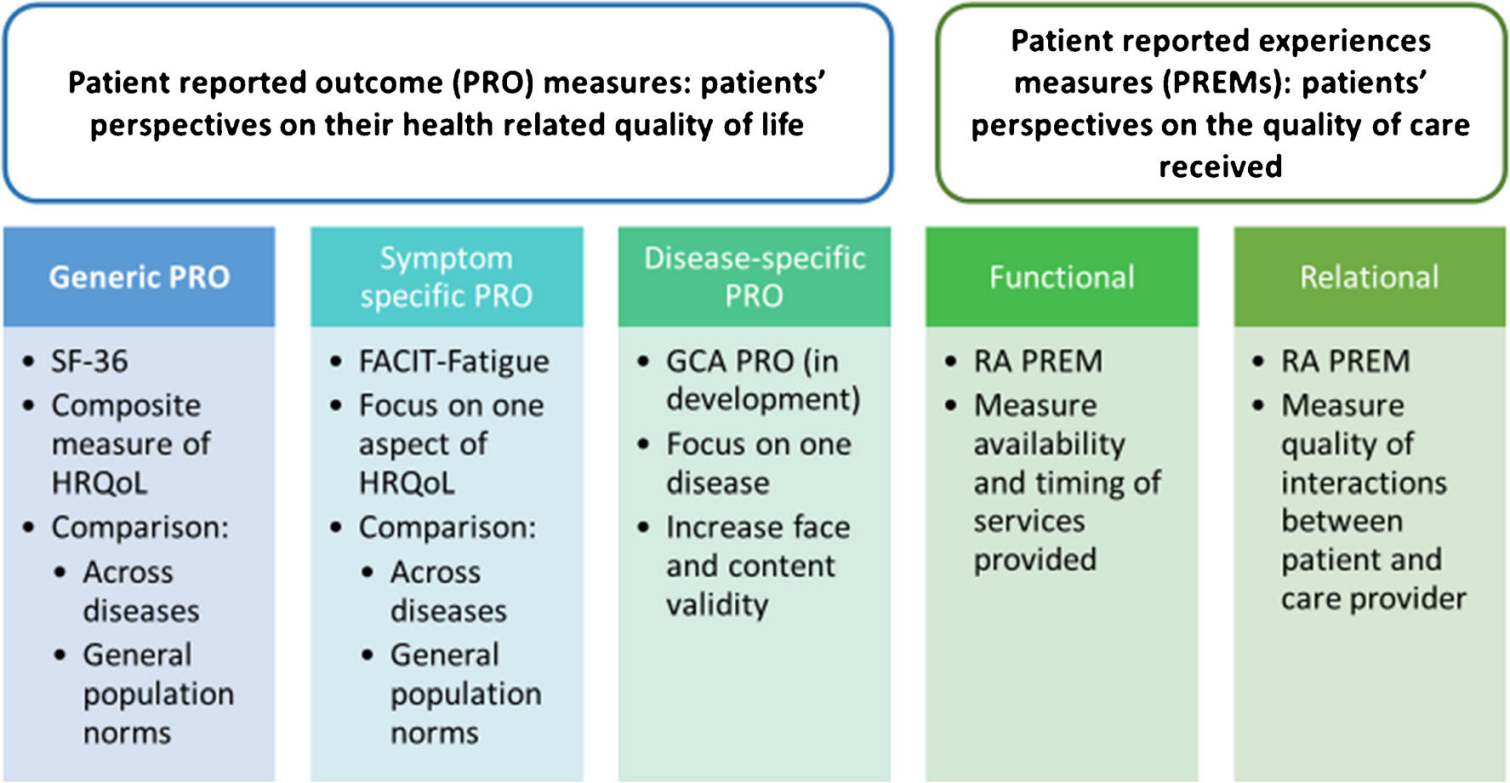
**Overview of patient reported outcome (PRO) measures and patient-reported experiences measures (PREMs).** SF-36: Short-form 36; FACIT-Fatigue: Functional Assessment of Chronic Illness Therapy-Fatigue scale; GCA PRO: Patient reported outcome for people with giant cell arteritis; RA PREM: patient reported experience measure for people with rheumatoid arthritis and other rheumatic diseases

The development of disease-specific PROs mandates inclusion of people with the disease in question at every stage, in line with FDA guidance [25]. Figure 1 illustrates the different stages of PRO development which can be used to develop disease-specific, generic, or symptom-specific PROs. Underpinning qualitative research to explore the full range of impacts of the disease and its treatment to people is performed first to ensure face and content validity of the final disease-specific PRO [26]. Themes important to people with the disease are then used as the basis for candidate questionnaire items, including stems and response categories. These are then piloted with cognitive interviewing techniques to test understanding, readability, and relevance [27]. Statistical analysis on data from a large-scale survey to test the PRO questions is then used to determine the final composition of the PRO. Exploratory Factor analysis [28] and Rasch analysis [29] are used to confirm the ideal structure of the PRO, and tests of validity are performed (e.g., test-retest and know groups validity) [30, 31]. Disease-specific PROs provide different information to generic and symptom-specific PROs and are therefore complimentary and can be used together [32]. See Fig. 2 for comparison of generic versus disease specific PROs.

### Disease-Specific PROs in Large Vessel Vasculitis?

The Outcome Measurement in Rheumatology (OMERACT) is an international collaborative initiative including researchers, clinicians, patient research partners, methodologists, pharmaceutical industry, and FDA representatives, working to define core sets of outcome measurements for use in RCTs, [33].

The OMERACT Vasculitis Working Group published a core set of domains and outcome measures for use in clinical trials in LVVin 2017 [34••] and highlighted the lack of a disease-specific PRO for people with GCA [34••, 35] and TAK [36]. The OMERACT group proposed a draft core set of domains for both GCA and TAK, including organ function, arterial function, biomarkers, fatigue, pain, and death and two additional preferred domains including psychosocial impact and physical function, plus separate additional GCA- and TAK- specific domains. A large-scale validation survey of a PRO for patients with GCA is currently underway in the UK, based on underpinning qualitative work in the UK and Australia [37]. Patient interviews and focus groups in the USA and Turkey have also identified domains of importance to patients with TAK [38], including “Pain and Discomfort,” “Fatigue and Low Energy Levels,” and “Emotional Effects”; these domains could underpin the development of a future disease-specific PRO for TAK [38].

#### How Can we Measure the Patient Experience of Care in Large Vessel Vasculitis?

PROs measure peoples’ perceptions of their health-related quality of life, whereas patient-reported experience measures (PREMs) are questionnaires that focus on the experience of receiving care. PREMs can be used as an indicator of quality of care and could be functional (e.g., what facilities were available and were they available in a timely way?) and/or relational (e.g., did you feel listened to and included in discussions about your treatment?) [39]. PREMs should also be underpinned by patient stakeholder involvement at each stage, as described in the development and validation of a PREM for patients with rheumatoid arthritis [40]. At present there is no PREM for either GCA or TAK, but this could be an important project for the future, particularly in view of the substantial variability and delays involved in getting a diagnosis [2]. A PREM may also be used to compare fast-track and conventional care strategies. See Fig. 2 for overview of difference between PROs and PREMs.

### What Is the Future for PROs for Large Vessel Vasculitis?

There is an ongoing need for generic, symptom, and disease-specific PROs for use in future clinical trials and observational studies inLVV. We know that people with vasculitis and their physicians rank outcomes of importance in different ways [41]; PROs are an important way of ensuring the patient perspectives are always included. Glucocorticoids have been the mainstay of treatment for both GCA and TAK, with the impact on people taking these medications quite significant [15]. A novel treatment-specific PRO to measure impact of glucocorticoids on people with rheumatic disease is also currently underway, led by the OMERACT Glucocorticoid working group [42]. This will complement the Glucocorticoid Toxicity Index (GTI), which is a clinician derived tool and not a PRO.

### Use of Individual Level PROs for Patient Care in Large Vessel Vasculitis? Future Opportunities?

Individual-level PROs can be used to monitor symptoms remotely, improve patient-clinician communication on important issues related to their health-related quality of life, and help promote self-management care [43••]. Using PROs within routine clinical care has been explored in other diseases, for example, the use of PROMIS symptom measures recorded on tablets prior to consultations in primary care; challenges included under documentation or under use of scores to change clinical practice, felt to be related to time constraints in primary care and lack of clinician support [44]. Qualitative analysis of a trial using daily completion of the Remote Monitoring of RA smartphone app over 4 weeks (patients reminded each day with a buzzer) with a physician review at the end of the 4 weeks, identified that patients reported that they felt their RA was “more visible” to clinicians and captured the bigger picture of their disease [45]. A year-long European study into use of E-PROs also found them to be acceptable to patients with RA, although some challenges, including lack of engagement by patients over the longer-term, particularly during periods of remission and if their clinicians did not act on the feedback provided were also identified [46]. There may be technical issues given that GCA patients may be an older age groups and therefore may not wish to complete PROs online. Patients with LVV may however wish to reduce the frequency of their face to face consultations because of the immunosuppressant effect of their diseases and treatments, impact of comorbidities such as cardiovascular disease and in the case of GCA, older age. Further work to explore the use of E-PROs and identify potential barriers and opportunities will need to be completed within these specific populations; it is likely that not all patients will feel the same about the use of EPROs and it will be important not to exclude any vulnerable groups due to their routine use in clinical practice.

## Conclusion

Generic PROs such as the SF-36 are already used routinely in clinical trials and observational studies in GCA and TAK to measure the impact on health-related quality of life. The development of disease-specific and treatment-specific PROs is underway. Future directions may include the use of E-PROs to facilitate remote consultations and the development of PREMs to allow measurement and improvement of care pathways for people with large vessel vasculitis.
